# Developmental changes in trak-mediated mitochondrial transport in neurons

**DOI:** 10.1016/j.mcn.2017.03.006

**Published:** 2017-04

**Authors:** Omar Loss, F. Anne Stephenson

**Affiliations:** School of Pharmacy University College London, 29/39 Brunswick Square, London WC1N 1AX, United Kingdom

**Keywords:** DIV, days in vitro, PSD-95, postsynaptic density protein 95, MAP, microtubule-associated protein 2, shRNA, short hairpin RNA, scrRNA, scrambled RNA, FITC, fluorescein isothiocyanate, EGFP, enhanced green fluorescent protein, DsRed, *discosoma* red fluorescent protein, DAPI, 4′,6-diamidino-2-phenylindole, PBS, phosphate-buffered saline, Mitochondrial transport, Trafficking kinesin binding protein, TRAK, Axonal transport, Kinesin

## Abstract

Previous studies established that the kinesin adaptor proteins, TRAK1 and TRAK2, play an important role in mitochondrial transport in neurons. They link mitochondria to kinesin motor proteins via a TRAK acceptor protein in the mitochondrial outer membrane, the Rho GTPase, Miro. TRAKs also associate with enzyme, O-linked *N*-acetylglucosamine transferase (OGT), to form a quaternary, mitochondrial trafficking complex. A recent report suggested that TRAK1 preferentially controls mitochondrial transport in axons of hippocampal neurons whereas TRAK2 controls mitochondrial transport in dendrites. However, it is not clear whether the function of any of these proteins is exclusive to axons or dendrites and if their mechanisms of action are conserved between different neuronal populations and also, during maturation. Here, a comparative study was carried out into TRAK-mediated mitochondrial mobility in axons and dendrites of hippocampal and cortical neurons during maturation in vitro using a shRNA gene knockdown approach. It was found that in mature hippocampal and cortical neurons, TRAK1 predominantly mediates axonal mitochondrial transport whereas dendritic transport is mediated via TRAK2. In young, maturing neurons, TRAK1 and TRAK2 contribute similarly in mitochondrial transport in both axons and dendrites in both neuronal types. These findings demonstrate maturation regulation of mitochondrial transport which is conserved between at least two distinct neuronal subtypes.

## Highlights

• Mitochondrial transport and velocity changes during neuronal maturation.
• TRAK1 and TRAK2 contribute to transport in axons and dendrites of immature neurons.
• In mature neurons TRAK1 controls axonal mitochondrial transport.
• In mature neurons TRAK2 controls dendritic mitochondrial transport.

## Graphical abstract

**Figure:**
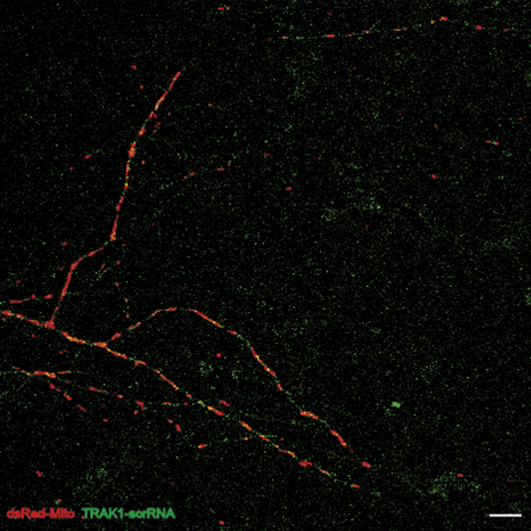

## 1 Introduction

Despite the brain being only 2% of the body weight, it consumes 20% of the body's resting energy. A typical neuron consumes ~ 4.7 million ATP molecules per second to power various brain functions such as neuronal survival, axonal growth and branching, generation of action potentials and synaptic transmission (Attwell and Laughlin, 2001, Zhu et al., 2012). Mitochondria are the cellular organelles that generate the ATP. For efficiency, they must be located adjacent to the sites requiring energy. It is known that mitochondria travel from their site of synthesis, the soma, along the axons and dendrites of neurons (anterograde movement) or vice versa (retrograde movement) to satisfy the energy demand essential for neuronal function. Indeed, defective mitochondrial trafficking and impaired mitochondrial function are increasingly implicated in neurological diseases (reviewed in (Chan, 2006, Mattson et al., 2008).

At any given time ~ 20–50% mitochondria in neurons are mobile (Brickley and Stephenson, 2011, Bros et al., 2015, Cai et al., 2005, Chang and Reynolds, 2006, Chang et al., 2006, Chen and Sheng, 2013, Jimenez-Mateos et al., 2006, Ligon and Steward, 2000, MacAskill et al., 2009a, Morris and Hollenbeck, 1993, Nguyen et al., 2014, Overly et al., 1996, Pekkurnaz et al., 2014, Ruthel and Hollenbeck, 2003, Trushina et al., 2012, van Spronsen et al., 2013, Wang and Schwarz, 2009). Mitochondrial transport in neurons has been shown to undergo maturation changes. For example in early studies, their movement in axons of growing neurons showed changes in the percentages of moving mitochondria with differences in anterograde versus retrograde transport. These observed changes were dependent on the length of the axons (Morris and Hollenbeck, 1993, Ruthel and Hollenbeck, 2003). More recently, Chang and Reynolds (2006) found that mitochondrial mobility in cortical neurons is greater in young neurons (5 days in vitro, DIV) compared to mature neurons (14 DIV) despite them having mitochondria with similar functionality properties i.e. their ability to buffer Ca^2 +^ and their membrane potential. Mitochondrial transport has been reported to differ between axons and dendrites of maturing hippocampal neurons. In axons, mitochondrial velocity was higher compared to in dendrites (Ligon and Steward, 2000, Overly et al., 1996).

Anterograde and retrograde movement of mitochondria in axons and dendrites is microtubule based. It is now generally accepted that the majority of transport is mediated by a quaternary complex of proteins. This complex is composed of the motor proteins, kinesin or dynein; the trafficking kinesin adaptor proteins (TRAKs); the TRAK acceptor protein, the outer mitochondrial membrane Rho GTPase, Miro, and the post-translational modification enzyme, *N*-acetylglucosamine transferase (OGT) (reviewed in (Schwarz, 2013, Stephenson and Brickley, 2011).

The family of TRAK proteins was initially identified in humans as the homologues of the *Drosophila melanogaster* gene product, Milton (Brickley et al., 2005, Stowers et al., 2002). Whereas *Drosophila* carries one Milton gene, mammals have two encoding TRAK1 and TRAK2. Decreased TRAK1 and TRAK2 expression and also the use of a TRAK2 dominant negative to inhibit the formation of the quaternary complex, leads to a decrease in mitochondrial mobility in hippocampal neurons (Brickley and Stephenson, 2011). The TRAK mitochondrial trafficking complex is also regulated by Miro and OGT. Both over-expression and down-regulation of Miro affect the transport of mitochondria in dendrites of hippocampal neurons (Macaskill et al., 2009b). Further, increases in Ca^2 +^ concentration alter the protein-protein binding properties of Miro and kinesin resulting in the inhibition of mitochondrial transport via dissociation of the trafficking complex (MacAskill et al., 2009a, Macaskill et al., 2009b). Increased levels of extracellular glucose decrease mitochondrial movement in axons of hippocampal neurons as a consequence of activation of OGT (Pekkurnaz et al., 2014).

A recent report suggested that TRAK1 and TRAK2 have potentially distinct roles in mitochondrial transport in different neuronal subcellular compartments since immunocytochemical studies revealed that TRAK1 was prevalently localized in axons whereas TRAK2 was more abundant in dendrites (van Spronsen et al., 2013). More support for this premise was that TRAK1-shRNA gene knockdown resulted in a decrease in mitochondrial mobility in axons (Brickley and Stephenson, 2011, van Spronsen et al., 2013) but in contrast, TRAK2-shRNA gene knockdown had no effect on axonal mitochondrial transport (Brickley and Stephenson, 2011) but van Spronsen et al. (2013) found that it impaired dendritic mitochondrial mobility. A subsequent investigation into TRAK1/2 subcellular distribution found a similar predominantly axonal distribution for TRAK1 and a dendritic distribution for TRAK2 (Loss and Stephenson, 2015). However, the demarcation between axonal versus dendritic distribution was not as evident as described by van Spronsen et al. (van Spronsen et al., 2013). A key difference between these two reports was that the study of van Spronsen et al. (2013) used 14 DIV hippocampal neurons whereas that of Loss and Stephenson (2015) used 6 DIV hippocampal neurons. A direct comparison of the findings between the two groups is therefore not tenable since there may be important maturation differences in mitochondrial transport at distinct stages of maturation. To address this, we have performed a systematic, comparative study in which the properties of TRAK-mediated mitochondrial transport were investigated in two different types of cultured primary neurons during maturation. The results are reported herein.

## 2 Materials and methods

### 2.1 Constructs and antibodies

The plasmids pDsRed1-Mito, pGreenTRAK1scrRNA (TRAK1-scrRNA), pGreenTRAK1shRNA (TRAK1-shRNA), pGreenTRAK2scrRNA (TRAK2-scrRNA) and pGreenTRAK2shRNA (TRAK2-shRNA) were as described previously (Brickley and Stephenson, 2011, Loss and Stephenson, 2015).

The following antibodies were used: rabbit polyclonal anti-TRAK1 antibodies (973–988), generated as described by Loss and Stephenson (2015); sheep anti-TRAK2 (874–889) antibodies, generated as described by Brickley et al. (2005); mouse monoclonal anti-tau (TAU-5), (RRID:AB_1603723, Abcam, Cambridge, UK, Cat. N. ab80579); mouse monoclonal anti-microtubule associate protein-2 (MAP-2), (RRID:AB_297885, Abcam, ab11267); mouse monoclonal anti-β-actin (RRID:AB_722536, Abcam ab40864); rabbit polyclonal anti-PSD95 (RRID:AB_444362, Abcam, ab18258), mouse monoclonal anti-synaptophysin [SY38] (RRID:AB_2198854, Abcam, ab8049); goat polyclonal anti-mouse IgG1 secondary antibody, Alexa Fluor 633 conjugate (RRID:AB_2535768, Thermo Fisher Scientific, Waltham, MA USA, A-21126); goat polyclonal anti-mouse IgG (H + L) secondary antibody, Alexa Fluor 594 conjugate (RRID:AB_2534073, Thermo Fisher Scientific, A-11005); goat polyclonal anti-rabbit IgG (H + L) secondary antibody, Alexa Fluor 594 conjugate (RRID:AB_2534095, Thermo Fisher Scientific, A-11037); goat polyclonal anti-mouse IgG (H + L) secondary antibody, Alexa Fluor 488 conjugate (RRID:AB_2534069, Thermo Fisher Scientific, A-11001).

### 2.2 Culturing and transfection of hippocampal and cortical neurons

Cultures of rat hippocampal and cortical neurons were prepared at a density of ~ 30,000 cells/cm^2^ on poly-d-lysine- (1 μg/ml) and laminin (2 μg/ml)-coated glass bottom culture dishes (P35G-1.5-14-C, Mattek Corporation, US) from hippocampi or cerebral cortices dissected from E18 rat embryos by standard methods (Goslin et al., 1998). Cultures were grown for 4–12 days in complete neurobasal media which was neurobasal media (Life Technologies) containing a 1 in 50 dilution of B27 (Life Technologies), 0.5 mM GlutaMax (Life Technologies), 0.4% (w/v) glucose and 1 × penicillin/streptomycin. Transfection of neurons was performed at 3–4, 7–8 or 11–12 days in vitro (DIV) using the calcium phosphate method. In brief, 48 h prior to transfection, neurobasal complete medium with antibiotics was removed and stored at 37 °C in 5% CO_2_ and replaced with fresh neurobasal complete medium. The transfection reaction was performed by addition of 6 μg EndoFree plasmid DNA at a ratio of 1:1 (pDsRed-Mito + pGreenTRAK1scrRNA, pGreenTRAK1shRNA, pGreenTRAK2scrRNA or pGreenTRAK2shRNA) to a solution containing 0.25 M CaCl_2_. This was added to an equal volume of 2 × HEPES-buffered saline, pH 7.14, and incubated at room temperature for 25 min. The transfection mixture was added to the neurons dropwise and incubated at 37 °C in 5% CO_2_ for 30 min. The media was aspirated and the neurons were washed twice with fresh neurobasal media prior to addition of the previously removed media. Live imaging and subsequent fixation of neurons with 4% (w/v) paraformaldehyde was carried out 48–72 h after transfection.

Cultures were analyzed from 3 different maturation stages, i.e. 6 DIV, 10 DIV and 14 DIV to encompass the full range of previous reports on TRAK-mediated mitochondrial trafficking (Brickley and Stephenson, 2011, Loss and Stephenson, 2015, van Spronsen et al., 2013).

### 2.3 Immunoblotting

Cell lysates were prepared using 1 × RIPA solubilisation buffer (50 mM Tris pH 8.0, 150 mM NaCl, 0.1% [v/v] SDS, 0.5% [v/v] sodium deoxycholate, 1% [v/v] Triton X-100, 1 mM phenylmethylsulphonyl fluoride, 0.5 mg/ml protease inhibitor cocktai. Immunoblotting was carried out exactly as described by Brickley et al. (2005) using final antibody concentrations of 1 μg/ml anti-TRAK1 (973–988) and 2 μg/ml anti-TRAK2 (874–889).

### 2.4 Immunocytochemistry

Neurons fixed with 4% (w/v) paraformaldehyde in glass bottom culture dishes were permeabilized and blocked with a solution containing 0.2% (v/v) Triton X-100 (Sigma, UK) and 10% (v/v) foetal bovine serum in PBS for 1 h at room temperature. Primary antibodies were diluted in the above solution, added to the cells and incubated overnight at 4 °C. Cells were washed three times with PBS (137 mM NaCl, 2.7 mM KCl, 10 mM Na_2_HPO_4_, 1.8 mM KH_2_PO_4,_ pH 7.4) containing 0.1% (v/v) Tween 20 (Sigma, UK) for 10 min with gentle rotation. Secondary antibodies were added at a dilution 5 times the concentration of the respective primary antibodies and incubated for 1 h at room temperature. Dishes were washed 3 × with PBS containing 0.1% (v/v) Tween 20 for 10 min with gentle rotation prior to fixation with 2% (w/v) paraformaldehyde for 5 min at room temperature. After washing a further 3 × in PBS, 10 μl fluorescence mounting solution (Dako, Agilent Technologies, Stockport, UK), containing 4′,6-diamidino-2-phenylindole (DAPI) for the visualization of nuclei was added to each dish and the area covered with a glass coverslip.

### 2.5 Confocal microscopy and image analysis

Confocal microscopy imaging was carried out using a Zeiss LSM 710 confocal microscope with oil immersion 40 × or 63 × objectives with sequential acquisition setting. EGFP and Alexa-Fluor 488 were excited with λ = 488 nm at 2% of intensity. DsRed and Alexa Fluor 594 were excited with λ = 561 nm at 2% intensity. Alexa-Fluor 633 was excited with λ = 633 nm at 2% of intensity. *Z*-stacks of neurons were taken at a resolution of 1024 × 1024 pixels (7.73 pixel/μm) with the pinhole at a setting of 2 μm using the ZEN light blue software (Zeiss, RRID:SCR_013672) ensuring that image saturation was not reached. Maximum projection intensities were generated and images were analyzed using ImageJ (NIH, USA, RRID:SCR_001935).

### 2.6 Determination of the efficiency of TRAK1 and TRAK2 shRNA knockdown in hippocampal and cortical neurons

To determine the efficiency of TRAK1 and TRAK2 knockdown in hippocampal and cortical neurons by the respective shRNAs, images from the Loss and Stephenson (2015) were utilised. Thus the endogenous levels of TRAK1 and TRAK2 were determined after treatment with pDsRedTRAK1scrRNA, pDsRedTRAK1shRNA, pGreenTRAK2scrRNA, or pGreenTRAK2shRNA (Loss and Stephenson, 2015). This was achieved by analysing the fluorescence intensity levels of TRAK1 and TRAK2 immunoreactivities after treatment with either the respective scrRNA or shRNA. The images used for analysis were taken from Fig. 3, Fig. 4 (Loss and Stephenson, 2015). For each image, the intensity of the TRAK1 or TRAK2 antibody staining, in both axons and dendrites of hippocampal and cortical neurons, treated with the specific shRNA (*n* = 3) was measured using ImageJ software and compared to the samples treated with the scrRNA (*n* = 3). The percentage of TRAK1 and TRAK2 knockdown levels was determined by comparing the intensity levels of samples treated with scrRNA versus samples treated with shRNA.

### 2.7 Live imaging of mitochondria

**Fig. 1:**
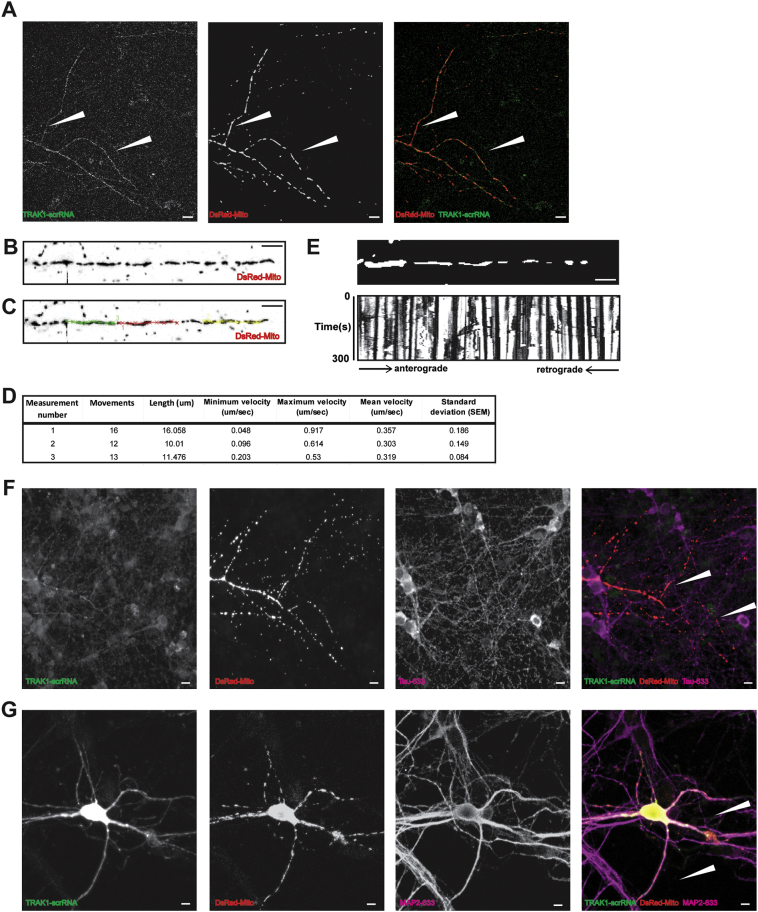
Methodology used for the analysis of mitochondrial mobility. A, single confocal images of hippocampal neurons expressing the mitochondrial marker, DsRed-Mito (red), and TRAK1-scrRNA (green). White arrows depict the neuronal processes considered during the analysis. B, a neuronal process from image A inverted and straightened using the ImageJ plugin, Straighten. C, an example of tracking of moving mitochondria from B above using the imageJ plugin, MTrackJ. D, an example of the data obtained from the mitochondrial movement tracking performed in C above. The table shows the number of movements each mitochondria made, the total length of movement, minimum, maximum and mean mitochondrial velocities ± SEM. E, resulting movie generated by the plugin MTrackJ and used for the generation of kymographs. F, image A after recording of mitochondrial movement, fixation and staining using anti-Tau antibodies (magenta) to distinguish axonal processes. F, an example of an image after live recording, fixation and staining using anti-MAP-2 antibodies (magenta) to distinguish dendritic processes. Scale bar = 10 μm. (For interpretation of the references to colour in this figure legend, the reader is referred to the web version of this article.)

Live confocal imaging of mitochondria was carried out using a Zeiss LSM 710 oil immersion 40 × objective with sequential acquisition setting 48–72 h post-transfection. Neurons were maintained for imaging in complete neurobasal media at 37 °C in 5% CO_2_. Transfected cells were identified under UV light with the fluorescein isothiocyanate (FITC) filter to enable visualization of the green fluorescence of the respective reporter followed by visualization under UV light with the rhodamine filter to check for the presence of DsRed-Mito. Neurons in culture were heterogeneous however, care was taken such that those selected for the analysis of mitochondrial transport had similar characteristics with regard to axonal and dendritic length, their number of branches and their observed viability. Anterograde mitochondria were those moving away from the soma. Retrograde mitochondria were those moving towards the soma. Bidirectional mitochondria were those moving towards or away from the soma for a portion of the recording and then switching to the opposite direction for the remaining time. For the identified transfected neuron, live imaging was carried out using λ = 561 nm excitation at 2% of the intensity of the diode-pumped solid-state laser to minimize bleaching and damage to neurons. Images of 512 × 512-pixel resolution (4.92 pixel/μm) with the pinhole at a setting of 3 μm were taken at 3 s intervals for 100 frames in a single focal plane. The short interval was used to minimize laser-induced neuronal damage. A mark on the glass bottom dish was made and a single confocal image of the region of interest was always taken using the FITC and rhodamine filters after the recording of the movie (Fig. 1A). This enabled the recognition of the imaged neuron after fixation and antibody staining with either anti-Tau, RRID:AB_1603723 (Fig. 1F) or anti-MAP-2, RRID:AB_297885 antibodies (Fig. 1G). The polarity of the axon or dendrite was determined by tracing the axon back to the cell body. The section of the axon/dendrite selected was at least 20 μm from the cell body, and contained at least eight mitochondria.

Note that all videos were recorded blind, i.e. the identification of the subcellular compartment was carried out post imaging and the maturation age was also revealed post-imaging.

### 2.8 Analysis of live imaging of mitochondria

After the live imaging of mitochondria, the images were opened using ImageJ (NIH, USA, RRID:SCR_001935) and exported as an AVI movie (Supplementary Video 1). Neuronal processes co-expressing the DsRed-Mito alone or with either TRAK1-shRNA, TRAK1-scrRNA, TRAK2-shRNA, TRAK2-scrRNA were analyzed. The chosen neuronal process was straightened using the imageJ plugin Straighten (Kocsis et al. 1991) (Fig. 1B and Supplementary Video 2) and the moving mitochondria were tracked using the imageJ plugin MTrackJ (Meijering et al. 2012) (Fig. 1C–E). Moving mitochondria are defined as those that are displaced > 2 μm from one site. This allowed the tracking of the movement of each moving mitochondria within the chosen sample together with the distance travelled, the direction (anterograde or retrograde) and the minimum, maximum and mean velocity (Fig. 1D). Following tracking of mitochondrial movements using MTrackJ a kymograph was generated (Fig. 1E). The analyzed samples were then fixed and stained using either the anti-Tau, RRID:AB_1603723 or anti-MAP-2, RRID:AB_297885 antibodies as described in live imaging of mitochondria (Fig. 1F–G). The imaged subcellular component was annotated as an axon or dendrite according to the co-staining given by anti-Tau, RRID:AB_1603723 and anti-MAP-2, RRID:AB_297885 antibodies.

For each condition and neuronal type, ~ 1000 mitochondria from ~ 30 neurons from 5 independent neuronal cultures were analyzed.

### 2.9 Statistics

Statistical analyses were performed using GraphPad Prism software. Unless otherwise stated, Kruskal-Wallis test was performed on all groups of data followed by a Tukey's range test for multiple comparisons.

## 3 Results

### 3.1 Detection of functional synapses in maturing cortical and hippocampal neurons

**Fig. 2:**
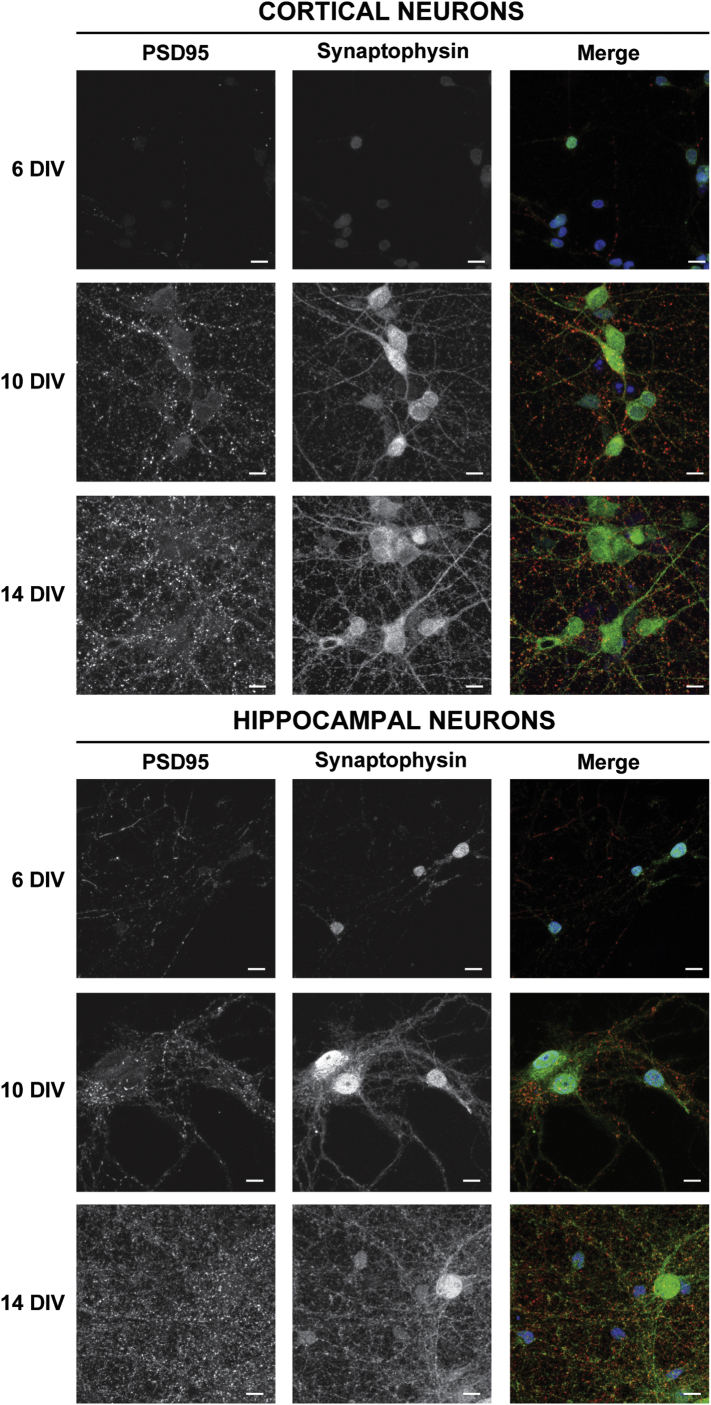
Determination of the maturation stages of hippocampal and cortical neurons by immunostaining with pre- and post-synaptic markers. Maximum projection intensities of cortical (top panels) and hippocampal (bottom panels) neurons at 6, 10 and 14 DIV co-stained with antibodies anti-synaptophysin (green) or anti-PSD-95 (red). Merged images are also shown. Nuclei were visualized using DAPI (blue). Confocal images were all taken at the same laser, gain and offset intensity. Images are representative of three separate cultures. Scale bar = 10 μm. (For interpretation of the references to colour in this figure legend, the reader is referred to the web version of this article.)

In order to determine the maturation stage of cultured primary hippocampal and cortical neurons as defined by the number of active synapses, the cultured neurons were co-immunostained for the pre-synaptic vesicle protein, synaptophysin, and the post-synaptic scaffolding protein, PSD-95, at 6, 10 and 14 DIV (Fig. 2). These time points were selected to represent the different maturation stages and also since most studies investigating mitochondrial trafficking have used cultured neurons at 6–14 DIV. Both synaptophysin and PSD-95 immunoreactivities were detectable as early as 6 DIV in both hippocampal and cortical neurons. However, the amount and the intensity of fluorescence were minimal and confined primarily to the cell body of the neurons. In addition, no co-localization (see merged images) between synaptophysin and PSD-95 was detected. In 10 DIV neurons, synaptophysin and PSD-95 immunoreactivities were both expressed at higher levels compared to 6 DIV. The respective immunostainings were distributed homogenously along axons and dendrites and both proteins were present in axons and dendrites simultaneously suggesting presence of both pre- and post-synaptic structures. In both 14 DIV cortical and hippocampal neurons the intensity and staining were even higher and no axons or dendrites that did not express PSD-95 and synaptophysin were detectable (Fig. 2). This is consistent with previous reports of the maturation of synaptic contacts in neurons in culture (Chang and Reynolds, 2006, Weiss et al., 1986). For these reasons the cultured neurons were named as young neurons for 6 DIV, maturing neurons for 10 DIV and mature neurons for 14 DIV. This nomenclature is in accordance with previous reports showing that neurons start differentiating immediately after plating and reach peak activity and expression of key neuronal features between 6 and 14 DIV (Aksenova et al., 1999, Craig et al., 1993, Dotti et al., 1988, Ichikawa et al., 1993, Zona et al., 1994).

### 3.2 Analysis of mitochondrial mobilities and velocities in axons and dendrites of primary neurons during maturation

**Fig. 3:**
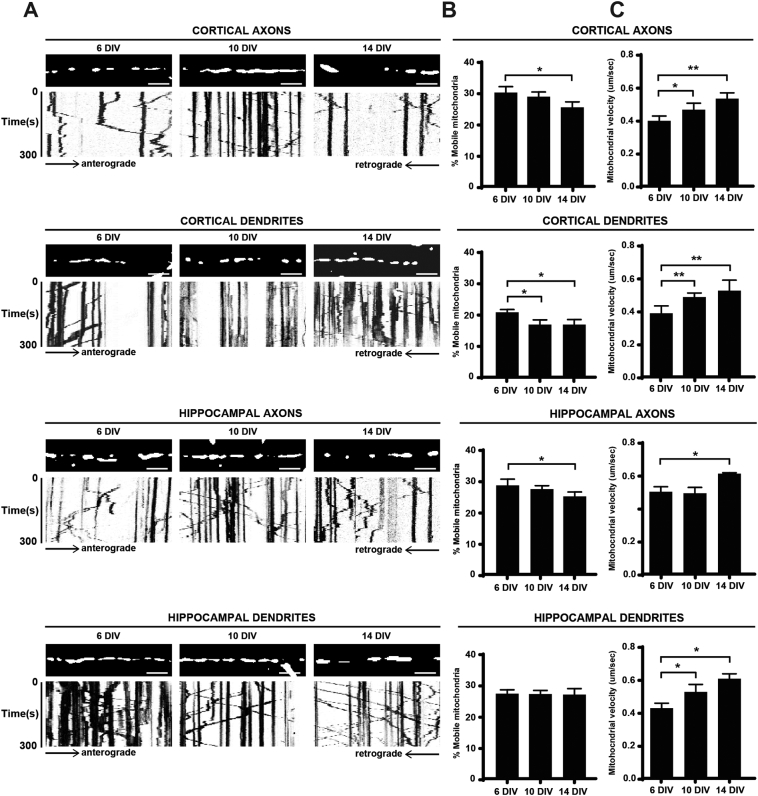
Analysis of mitochondrial mobility and velocity in axons and dendrites of cortical and hippocampal neurons during maturation. A, representative kymographs of time-lapse images of moving mitochondria in cortical axons, cortical dendrites, hippocampal axons and hippocampal dendrites expressing the mitochondrial marker DsRed-Mito in young (6 DIV), maturing (10 DIV) and mature (14 DIV) neurons. B, C, histograms showing the percentage of mobile mitochondria (B) and the mitochondrial mean velocities (C) at the different maturation stages. The results for each neuronal type and condition are the mean of ~ 1000 mitochondria ± SEM from ~ 30 neurons from 5 independent neuronal cultures.

Initially, the properties of mitochondrial transport, i.e. the percentage of mobile mitochondria, their direction (anterograde or retrograde) and the mitochondrial mean velocities in axons and dendrites of two neuronal cell types, (hippocampal and cortical neurons) at 6, 10 and 14 DIV, were determined. The results are summarized in Table 1, Table 2 and Fig. 3 with representative examples of live imaging shown in Supplementary Video 1, Supplementary Video 2.

It was observed that the percentage of mobile mitochondria in cortical axons, hippocampal axons and hippocampal dendrites at all maturation stages were similar and in the range ~ 25–30%. However in cortical dendrites, the percentage mobility was lower and in the range of 17–20% (Table 1, Fig. 3B). In axons and dendrites of cortical neurons and in axons of hippocampal neurons there was a trend to a decrease in the percentage of mobile mitochondria with maturation. In cortical axons there was a significant decrease in the percentage of mobile mitochondria at 14 DIV, i.e. from 29.6% at 6 DIV to 25.1% at 14 DIV (Table 1, Fig. 3A–B). When normalized to 6 DIV, this corresponded to a 15% decrease in the percentage change of mobile mitochondria. In cortical dendrites, an equivalent significant decrease in the percentage of mobile mitochondria at 10 DIV and 14 DIV (20.5% versus 16.5%) was observed (Table 1, Fig. 3A–B). When normalized to 6 DIV, this corresponded to a 20% decrease in the percentage change of mobile mitochondria. In hippocampal axons a significant decrease in the percentage of mobile mitochondria during maturation was observed at 14 DIV (28.4% versus 24.9%) (Table 1, Fig. 3A–B). When normalized to 6 DIV, this corresponded to a 12% decrease in the percentage change of mobile mitochondria. In contrast, no significant changes were evident in hippocampal dendrites (Table 1, Fig. 3A–B).

The direction of moving mitochondria in both neuronal types and at all maturation stages showed an ~ 50–50 anterograde-retrograde distribution of mobile mitochondria. No major differences were seen between the different maturation stages with respect to changes in the percentage of anterograde, retrograde and bidirectional mitochondria (Table 2). However in hippocampal axons, there was a significant difference in the anterograde movement between 6 DIV and 10 DIV. Values were for anterograde transport, 49.9% of the total mobile mitochondrial population at 6 DIV compared to 44.2% at 10 DIV. In all samples analyzed, between 9.4% and 13.1% of the total mobile mitochondria were shown to move bidirectionally. However, no significant changes were found for the maturation stages considered (Table 2).

With respect to mitochondrial mean velocities, the results summarized in Table 1 and Fig. 3C show that the mean velocities in axons and dendrites of hippocampal neurons were between 0.43 and 0.61 μm/s whereas the mean velocities in axons and dendrites of cortical neurons were between 0.39 and 0.53 μm/s. Mitochondrial velocities in cortical neurons were 15%–20% lower than in hippocampal neurons.

It was evident that mean mitochondrial velocities increased with maturation. This was the case for mitochondria in axons and dendrites of both hippocampal and cortical neurons. In cortical axons and dendrites, respective significant increases from 0.39 μm/s to 0.53 μm/s were seen (Table 1, Fig. 3C). This is a 36% increase in velocity when compared to 6 DIV. Mitochondrial velocities significantly increased from 0.50 μm/s to 0.61 μm/s (a 22% increase in velocity compared to 6 DIV) in axons of hippocampal neurons whereas in hippocampal dendrites velocities increased from 0.43 μm/s to 0.61 μm/s (Table 1, Fig. 3C), a 42% increase in velocity compared to 6 DIV.

### 3.3 TRAK1 knockdown decreases mitochondrial movement in axons of cultured cortical and hippocampal neurons during maturation

In order to determine the effect of TRAK1 knockdown on axonal and dendritic mitochondrial movement, shRNA studies were performed using the previously characterized TRAK1-shRNA or the scrambled control, TRAK1-scrRNA, in axons and dendrites of hippocampal and cortical neurons at different stages of maturation (Brickley and Stephenson, 2011, Loss and Stephenson, 2015). The efficiency of endogenous TRAK1 knockdown in neurons treated with the respective TRAK1-shRNA was 75% and 86% in axons of hippocampal and cortical neurons respectively and, 32% and 61% knockdown in dendrites of hippocampal and cortical neurons respectively.

**Fig. 4:**
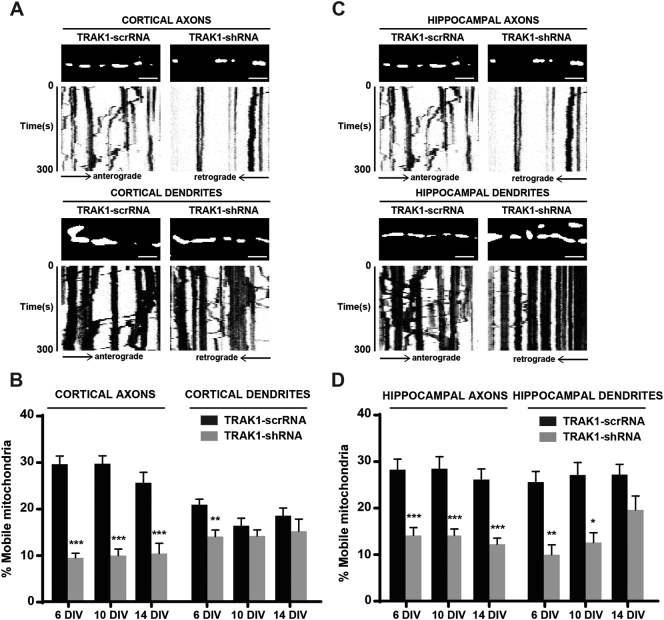
The effect of TRAK1-shRNA on mitochondrial mobility in axons and dendrites of cortical and hippocampal neurons during maturation. A, C, representative kymographs of time-lapse images of moving mitochondria in cortical (A) and hippocampal (C) axons and dendrites expressing the mitochondrial marker, DsRed-Mito, and either TRAK1-shRNA or TRAK1-scrRNA control sample in 6 DIV neurons. B, D, histograms showing the percentage of mobile mitochondria in axons and dendrites of cortical (B) and hippocampal (D) neurons at 6, 10 and 14 DIV. The results for each neuronal type and condition are the mean of ~ 1000 mitochondria ± SEM from ~ 30 neurons from 5 independent neuronal cultures.

The percentage of mobile mitochondria, their direction and mean velocities were analyzed and compared to neurons expressing the mitochondrial reporter, DsRed-Mito, alone. The results are summarized in Table 3, Table 4; in Fig. 4 where representative kymographs are shown and also in Supplementary Video 3, Supplementary Video 4. From Table 3, it can be seen that there were no essential differences between the TRAK1-scrRNA control samples and the neurons transfected with DsRed-Mito alone thus all further comparisons were made between TRAK1-shRNA and control, TRAK1-scrRNA samples.

TRAK1-shRNA knockdown resulted in the decrease of the percentage of mobile mitochondria with respect to TRAK1-scrRNA controls in axons of both cortical and hippocampal neurons at all stages of maturation (Table 3, Fig. 4A–D, Supplementary Video 3 (cortical) and Supplementary Video 4 (hippocampal). For cortical axons, the percentage of mobile mitochondria was 29.5% at 6 DIV, 29.6% at 10 DIV and 25.5% at DIV 14 in the TRAK1-scrRNA controls versus 9.4% at DIV 6, 9.9% at DIV 10 and 10.3% at DIV 14 in the TRAK1-shRNA samples (Table 3, Fig. 4A–B). For hippocampal axons the percentage of mobile mitochondria was 28.0% at 6 DIV, 28.2% at 10 DIV and 25.9% at DIV 14 in the TRAK1-scrRNA controls versus 13.9% at DIV 6 and DIV 10 and 12.0% at DIV 14 in the TRAK1-shRNA samples (Table 3, Fig. 4C–D). When normalized to TRAK1-scrRNA controls, these variations corresponded to a 50–68% decrease in the percentage change of mobile mitochondria.

In cortical and hippocampal dendrites a decrease in the percentage of mobile mitochondria was noticed primarily in the young and maturing neurons following TRAK1-shRNA treatment. Thus for dendrites of cortical neurons the decrease in the percentage of mobile mitochondria was only significant at 6 DIV, from 20.7% in controls to 13.9% in TRAK1-shRNA samples (Table 3, Fig. 4A–B). When normalized to TRAK1-scrRNA controls, this variation corresponded to a 33% decrease in the percentage change of mobile mitochondria. For dendrites of hippocampal neurons, the decrease in the percentage of mobile mitochondria was significant at 6 DIV and 10 DIV. Values were 25.5% at 6 DIV and 27.0% at 10 DIV for controls and 9.9% at 6 DIV and 12.5% at 10 DIV for TRAK1-shRNA samples (Table 3, Fig. 4C–D). When normalized to TRAK1-scrRNA controls, this variation corresponded to a 55% decrease in the percentage change of mobile mitochondria for both 6 and 10 DIV.

No major differences were seen between the controls and TRAK1-shRNA treated neurons with respect to the percentage of anterograde, retrograde and bidirectional mitochondria (Table 4). Similarly to non-treated neuronal cultures as described above, the direction of moving mitochondria in both neuronal types and at all maturation stages showed an ~ 50–50 anterograde-retrograde distribution of mobile mitochondria. Only in the case of hippocampal axons at 6 DIV was there a significant difference. An increase in the percentage of anterograde mitochondria from 39.8% to 44.5% of the total mobile mitochondria was found between the controls and TRAK1-shRNA treated neurons. In all cultures between 8.9%–12.2% of the total mobile mitochondria were shown to move bidirectionally. However, no significant changes were evident between controls and TRAK1-shRNA treated neurons (Table 4).

With regard to mitochondrial velocity, a gradual increase was seen in controls and TRAK1-shRNA-treated cultures similarly to that observed for neuronal maturation (Table 1). TRAK1-shRNA knockdown had no significant effect on mitochondrial velocities in axons and dendrites of cortical and hippocampal neurons during maturation (Table 3).

### 3.4 TRAK2 knockdown decreases mitochondrial movement in dendrites of cultured cortical and hippocampal neurons during maturation

Similarly to TRAK1, to determine the effect of TRAK2 knockdown on axonal and dendritic mitochondrial movement, shRNA studies were performed using the previously characterized TRAK2-shRNA or the scrambled control, TRAK2-scrRNA, in axons and dendrites of hippocampal and cortical neurons during maturation (Brickley and Stephenson, 2011, Loss and Stephenson, 2015). The efficiency of endogenous TRAK2 knockdown in neurons treated with the respective TRAK2-shRNA showed levels of 64% and 81% knockdown in axons of hippocampal and cortical neurons respectively and, 30% and 43% knockdown in dendrites of hippocampal and cortical neurons respectively.

**Fig. 5:**
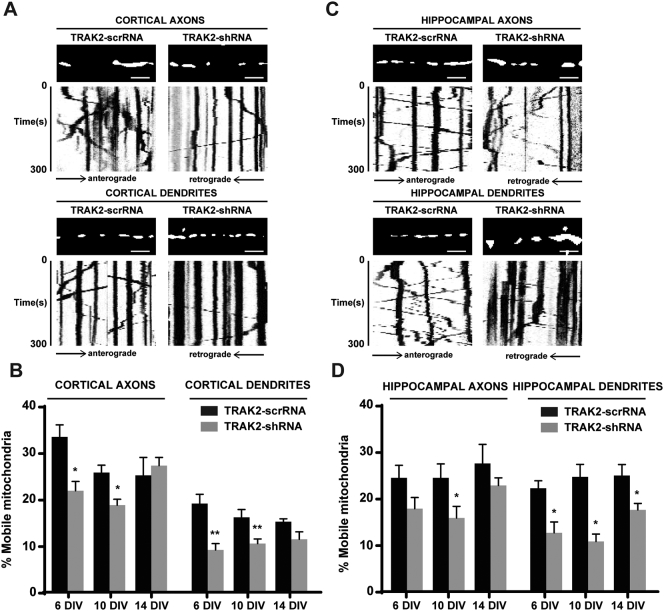
The effect of TRAK2-shRNA on mitochondrial mobility in axons and dendrites of cortical and hippocampal neurons during maturation. A, C, representative kymographs of time-lapse images of moving mitochondria in cortical (A) and hippocampal (C) axons and dendrites expressing the mitochondrial marker, DsRed-Mito, and either TRAK2-shRNA or TRAK2-scrRNA control sample in 6 DIV neurons. B, D, histograms showing the percentage of mobile mitochondria in axons and dendrites of cortical (B) and hippocampal (D) neurons at 6, 10 and 14 DIV. The results for each neuronal type and condition are the mean of ~ 1000 mitochondria ± SEM from ~ 30 neurons from 5 independent neuronal cultures.

The percentage of mobile mitochondria, their direction and mean velocities were analyzed and compared to neurons expressing the mitochondrial reporter, DsRed-Mito, alone. The results are summarized in Table 5, Table 6; in Fig. 5 where representative kymographs are shown and also in Supplementary Video 5, Supplementary Video 6. From Table 5, it can be seen that there were no essential differences between the TRAK2-scrRNA control samples and the neurons transfected with DsRed-Mito alone thus all further comparisons were made between TRAK2-shRNA and control, TRAK2-scrRNA samples.

TRAK2-shRNA knockdown resulted in the significant decrease of the percentage of mobile mitochondria with respect to TRAK2-scrRNA controls in axons and dendrites of both cortical and hippocampal neurons at all maturation stages except for 14 DIV axons of both neuronal types and 6 DIV axons of hippocampal neurons (Table 5, Fig. 5A–D, Supplementary Video 5 (cortical) and Supplementary Video 6 (hippocampal). In cortical dendrites, the percentage of mobile mitochondria was 18.8% at 6 DIV, 15.8% at 10 DIV and 14.9% at DIV 14 in the TRAK2-scrRNA controls versus 8.8% at DIV 6, 10.2% at DIV 10 and 11.1% at DIV 14 in the TRAK2-shRNA samples (Table 5, Fig. 5A–B). For hippocampal dendrites the percentage of mobile mitochondria was 22.3% at 6 DIV, 24.7% at 10 DIV and 25.0% at DIV 14 in the TRAK2-scrRNA controls versus 12.7% at DIV 6, 10.8% at DIV 10 and 17.6% at DIV 14 in the TRAK2-shRNA samples (Table 5, Fig. 5C–D). When normalized to TRAK2-scrRNA controls, these variations corresponded to a 25–56% decrease in the percentage change of mobile mitochondria.

With respect to axons, a decrease in the percentage of mobile mitochondria was noticed primarily in the young and maturing neurons following TRAK2-shRNA treatment, except for 6 DIV hippocampal axons. Thus for axons of cortical neurons the decrease in the percentage of mobile mitochondria was significant at 6 and 10 DIV. Values were 33.3% at 6 DIV and 25.6% at 10 DIV for controls versus 21.7% at 6 DIV and 18.6% at 10 DIV for TRAK2-shRNA samples (Table 5, Fig. 5A–B). When normalized to TRAK2-scrRNA controls, this variation corresponded to a 30% decrease in the percentage change of mobile mitochondria for both 6 DIV and 10 DIV. For axons of hippocampal neurons, the decrease in the percentage of mobile mitochondria was only significant at 10 DIV. Values were 24.5% for controls and 15.9% for TRAK2-shRNA samples (Table 5, Fig. 5C–D). When normalized to TRAK2-scrRNA controls, this variation corresponded to a 35% decrease in the percentage change of mobile mitochondria.

No major differences were seen between the controls and TRAK2-shRNA treated neurons with respect to the percentage of anterograde, retrograde and bidirectional mitochondria (Table 6). Similarly to non-treated neuronal cultures as described above, the direction of moving mitochondria in both neuronal types and at all stages of maturation showed an ~ 50–50 anterograde-retrograde distribution of mobile mitochondria. Only in the case of hippocampal axons at 10 DIV, a significant decrease in the percentage of anterograde mobile mitochondria (44.6% versus 39.9% of the total mobile mitochondria) and a consequent retrograde increase (43.0% versus 49.4% of the total mobile mitochondria) was noticed between the TRAK2-scrRNA control samples and the TRAK2-shRNA samples (Table 6). In all cultures, between 6.8% and 12.4% of the total mobile mitochondria were shown to move bidirectionally. Significant differences were seen between the control and TRAK2-shRNA samples only in 6 DIV cortical axons (decrease from 9.4% to 7.5%) and 6 DIV hippocampal axons (increase from 6.8% to 10.7%) (Table 6).

With regard to mitochondrial velocity, a gradual increase was seen in controls and TRAK2-shRNA-treated cultures similarly to that observed for neuronal maturation (Table 1). TRAK2-shRNA knockdown had no significant effect on mitochondrial velocities in axons and dendrites of cortical and hippocampal neurons during maturation (Table 5).

### 3.5 TRAK2 protein levels decrease during maturation

**Fig. 6:**
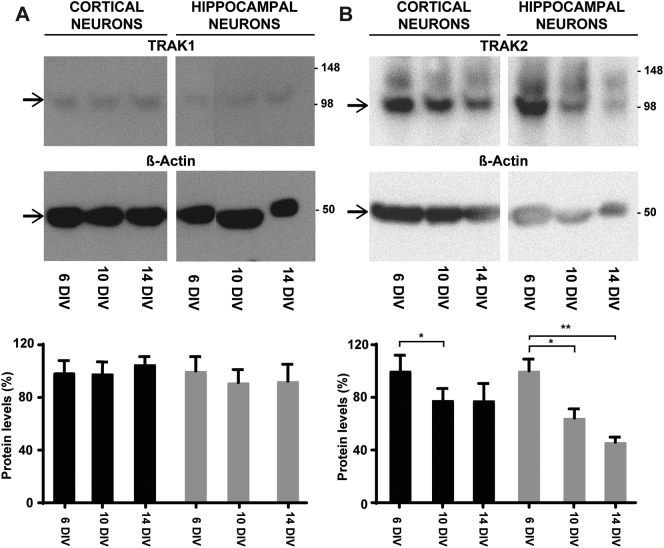
TRAK1 and TRAK2 protein expression levels during maturation. Cell lysates of 6, 10 and 14 DIV cortical and hippocampal neurons were prepared and analyzed by immunoblotting with anti-TRAK1 (973–988) (A) and anti-TRAK2 (874–889) (B) or anti-β-actin antibodies as described in Materials and methods. The specificity of the antibodies for TRAK1 and TRAK2 was described in Loss and Stephenson (2015); and Brickley et al. (2005), respectively. Numbers on the right of each immunoblot are molecular weights in kDa. Images are a representation of *n* = 2 independent experiments. Column graphs are the quantification of *n* = 2 immunoblots where protein levels are expressed as the percentage changes in respect to 6 DIV, normalized to the levels of β-actin. ^⁎^ρ < 0.05, ^⁎⁎^ρ < 0.01.

A possible explanation of the changes in specificity between axons and dendrites of TRAK in determining mitochondrial mobility is the expression levels of TRAK1 and TRAK2 during maturation. To investigate this, immunoblotting was carried out in order to determine the levels of TRAK1 and TRAK2 in cortical and hippocampal neurons at 6, 10 and 14 DIV. As shown, TRAK1 levels did not change significantly during maturation of both cortical and hippocampal neurons (Fig. 6A). In contrast, a significant decrease in the levels of TRAK2 was detected in both cortical and hippocampal neurons, in both 10 DIV and 14 DIV when compared to 6 DIV (Fig. 6B). Indeed, quantification of the protein levels detected by immunoblotting showed a 23% decrease of TRAK2 protein levels in both 10 and 14 DIV compared to 6 DIV in cortical neurons and a 36% and 54% decrease in 10 DIV and 14 DIV, respectively, in hippocampal neurons (Fig. 6B).

## 4 Discussion

In this paper a comparison of mitochondrial transport per se and TRAK-mediated mitochondrial trafficking in axons and dendrites of cortical and hippocampal neurons cultured in parallel and during maturation was made since no such comprehensive study has been previously carried out. In summary, a decrease in the percentage of mobile mitochondria in axons and dendrites of cortical and hippocampal neurons during maturation was found in contrast to mitochondrial velocity which was observed to increase during maturation. TRAK1- and TRAK2-shRNA knockdown revealed that in mature neurons, TRAK1 predominantly mediates axonal mitochondrial transport whereas dendritic transport is mediated via TRAK2. In young and maturing neurons, TRAK1 and TRAK2 participate similarly in mitochondrial transport in both axons and dendrites in both neuronal types. These findings thus demonstrate regulation of TRAK-mediated mitochondrial transport during maturation which is conserved between at least two distinct neuronal subtypes.

### 4.1 Mitochondrial movement changes during neuronal maturation

Over the past 20 years several groups have investigated mitochondrial transport in neurons. Changes in the percentages of moving mitochondria and their velocities were described both during neuronal maturation and between axonal and dendritic mitochondrial transport (Chang and Reynolds, 2006, Ligon and Steward, 2000, Morris and Hollenbeck, 1993, Overly et al., 1996, Ruthel and Hollenbeck, 2003). Notably, Chang and Reynolds (2006) found a decreased mitochondrial mobility in synaptically mature compared to synaptically immature neurons. Moreover, recent reports showed decreased mitochondrial mobility during maturation of both hippocampal (Obashi and Okabe, 2013) and cortical (Lewis et al., 2016) axons. In addition, Lewis et al. (2016) also demonstrated a progressive decrease in mitochondrial mobility both in vitro and in vivo.

Since the earlier cited publications, several proteins that mediate mitochondrial trafficking have now been identified, in particular the TRAK family of kinesin adaptor proteins amongst others (Brickley and Stephenson, 2011, Bros et al., 2015, Cai et al., 2005, Chang et al., 2006, Chen and Sheng, 2013, Jimenez-Mateos et al., 2006, MacAskill et al., 2009a, Nguyen et al., 2014, Pekkurnaz et al., 2014, Trushina et al., 2012, van Spronsen et al., 2013, Wang and Schwarz, 2009). However, no studies have yet compared TRAK-mediated mitochondrial mobilities and their velocities in cultured neurons at different stages of maturation and between different neuronal types in parallel. Thus here, we have analyzed mitochondrial mobilities, velocities and direction in axons and dendrites of cultured hippocampal and cortical young, maturing and mature neurons and the contribution of TRAK-mediated mitochondrial transport to these mechanisms.

In the literature, values reported for the percentage of mobile mitochondria at any one given time in axons and dendrites is between 20%–50% with their velocities ranging from 0.2–0.6 μm/s (see references above). This is the case across taxa since similar percentage mitochondrial mobilities and velocities have been described for the fly and frog nervous systems (Pilling et al., 2006, Zhang et al., 2010). The values reported herein fall within this range. But, notably, the percentage of mobile mitochondria decreased and their velocities increased during neuronal maturation. This decrease in mobility may be a result of the availability of motor proteins or the kinesin adaptors. The expression levels of any of the proteins known to be kinesin adaptors for mitochondrial transport such as TRAKs and syntabulin (Cai et al., 2005) would lead to a decreased or increased efficiency in the formation of the quaternary mitochondrial trafficking complex (Chen and Sheng, 2013). The increase in velocity with maturation is in contrast to the decreased mitochondrial mobility. Some mitochondria in mature neurons might express higher levels of Miro thus promoting the efficiency of the formation of the Miro/TRAK/motor protein complex which leads to increased mitochondrial velocity.

It was of note that the percentage of mobile mitochondria decreased in mature neurons despite the fact that mature neurons have 30–80-fold more active synapses than non-mature neurons and presumably a higher energy requirement (Cullen et al., 2010).

With respect to axonal versus dendritic transport of mitochondria, some reports found no change between the number of moving mitochondria in the two subcellular compartments (Ligon and Steward, 2000) whereas others found less mitochondrial mobility in dendrites compared to axons (Overly et al., 1996). The only difference in this study was a lower percentage of moving mitochondria in dendrites compared to axons in cortical neurons only. Differences between this study and published works may be explained by variations in methods for the detection of mobile mitochondria (Mitotracker versus DsRed-Mito) and the maturation stage of the hippocampal cultures.

One of the goals of this study was also to compare mitochondrial transport between cultured cortical and hippocampal neurons, since to our knowledge no such in parallel comparison has been previously been carried out. No significant differences were apparent for the percentages of moving mitochondria and their velocities between cortical and hippocampal neurons. In agreement, a recent study reported that the percentage of mobile mitochondria in primary cortical neurons was similar to previous published studies using hippocampal neurons (Nguyen et al., 2014).

It should be recognized that mitochondrial transport in cultured neurons is not necessarily reflective of mitochondrial mobility in vivo. Mitochondrial transport has been investigated in organotypic brain slices and in in vivo animal models. The percentage of mobile mitochondria and their velocities in organotypic brain slices are similar to that described for cultured neurons (Jackson et al., 2014, Ohno et al., 2011). In in vivo models, the percentage of mobile mitochondria was lower, in the range of 10–15% and the velocities were in the range, 0.7 μm/s–1.9 μm/s which is higher than found in cultured neurons (Lewis et al., 2016, Misgeld et al., 2007, Sajic et al., 2013). The data were obtained however from peripheral rather than central neurons from transgenic mouse lines in which mitochondrially-targeted fluorescent proteins are selectively expressed in neurons (Misgeld et al., 2007, Sajic et al., 2013).

Overall, mitochondrial movement along axons and dendrites varies during maturation in vitro. These changes may impact on the mitochondrial driven, Ca^2 +^-dependent, ATP supply during axonal growth and branching, generation of action potentials, and synaptic transmission.

### 4.2 TRAK-mediated mitochondrial transport in neurons

With respect to TRAK-mediated mitochondrial transport, a role for TRAK1 in axonal transport was confirmed with no significant differences observed at the different maturation stages analyzed. TRAK1-shRNA did however, also impair mitochondrial mobility in dendrites. This was most evident in young and maturing neurons and also in hippocampal versus cortical dendrites. These findings suggest that TRAK1 mediates mitochondrial trafficking in both axons and dendrites of young, maturing neurons but it then assumes a more specific axonal role once the neuron is mature. The latter is in agreement with van Spronsen et al. (2013) who reported that TRAK1 knockdown effected primarily axonal mitochondrial transport in 13 DIV mature neurons.

TRAK2 was also found to contribute to both axonal and dendritic mitochondrial transport since TRAK2-shRNA specifically and significantly inhibited mobility in these subcellular compartments. However, the percentage inhibition of transport was higher for dendrites compared to axons. Further, inhibition of axonal mitochondrial transport was more evident in young and maturing neurons. In mature neurons, TRAK2-shRNA inhibited only dendritic mitochondrial transport. Previous studies proposed a role for TRAK2 in dendritic mitochondrial transport in 13 DIV neurons based on TRAK2-shRNA studies (van Spronsen et al., 2013). This was in agreement with Stephenson and Brickley (2011) who found that TRAK2-shRNAs had no effect on mitochondrial mobility in axons albeit in younger (6 DIV) cultured hippocampal neurons. The contribution of TRAK2 to mitochondrial transport in axons in immature neurons as found here may possibly be explained by the analysis of at least two times the number of mitochondria analyzed in previous studies (Brickley and Stephenson, 2011, van Spronsen et al., 2013). The fact that TRAK2, but not TRAK1, protein levels decrease during maturation also suggests changes in the subcellular specificity of TRAK2 during maturation.

The respective functional roles of TRAK1 and TRAK2 are in agreement with their subcellular localizations. A prevalent axonal distribution for TRAK1 and dendritic localization for TRAK2 in mature neurons was described (van Spronsen et al., 2013). However, Loss and Stephenson (2015) found a less marked distinction in the distribution of TRAK1 and TRAK2 between axons and dendrites of immature neurons which again fit with their functional roles during maturation as described here. In here, we also show a decrease in TRAK2, but not TRAK1, protein levels during maturation. TRAK2 could play a more general role in developing neurons, controlling mitochondrial transport at all subcellular levels. With maturity, TRAK2 could be more confined to dendrites, whereas TRAK1 remains axonal, hence the differences in protein levels.

The functional roles of TRAK1 and TRAK2 in dendrites versus axons may be explained by their respective distributions but they may also be due at least in part, to the availability of the motor proteins. It is known that kinesin and dynein play important roles in selective trafficking into axons and dendrites (Kapitein and Hoogenraad, 2011). In axons, kinesin and dynein are both essential for the movement of vesicles but in dendrites, dynein alone is sufficient for vesicular transport (Kapitein et al., 2010, Zheng et al., 2008). In mature neurons, TRAK1 has been shown to bind both kinesin and dynein whereas TRAK2 binds exclusively dynein (van Spronsen et al., 2013). Whether this is the case in young, maturing neurons, still needs to be established. There are three kinesin 1 genes (KIF5A, KIF5B and KIF5C). All are expressed in different neuronal types and have different roles in neuronal maturation and function (Kanai et al., 2000). At least in adult brain, TRAKs are known to bind to KIF5A (Brickley et al., 2005) but the TRAK-kinesin binding properties might change during maturation. Indeed, TRAK1 and TRAK2 have been shown to bind with different affinities to the different KIF5 isoforms (Randall et al., 2013). Thus the availability of these motor protein isoforms in neurons during maturation, in addition to TRAK2 expression levels, could be a factor determining the fate of axonal versus dendritic TRAK distribution.

## 5 Conclusion

Disruption of mitochondrial transport has been implicated in several neurodegenerative diseases (Hirai et al., 2001, Praprotnik et al., 1996, Stokin and Goldstein, 2006, Terry, 1996). It is unclear if this dysfunction is causative or a consequence of other molecular or cellular changes associated with mechanisms of neurodegeneration. Different degenerative disorders target distinct neuronal populations thus it is important to have knowledge of basic mechanisms such as those involved in transporting mitochondria, in order to understand pathogenic changes. Here we have provided a comprehensive study of the properties of mitochondrial transport in two neuronal subtypes at different stages of maturation. This sets a strong foundation for future studies of mitochondrial trafficking in relation to neurodegenerative disease.

The following are the supplementary data related to this article.

Supplementary Video 1 An example of mitochondrial dynamics in axons of hippocampal neurons. Neurons were transfected with dsRed-Mito at 7 DIV. Time lapsed images were obtained of living neurons at 10 DIV capturing an image every 3 s for a total of 5 min (total images = 100). The video is shown at 7 frames per second (fps). Supplementary Video 1.

Supplementary Video 2 An example of straightened axons from Supplementary Video 1 for analysis using ImageJ plugin Straighten. The video is shown at 7 fps. Supplementary Video 2.

Supplementary Video 3 Mitochondrial mobility in axons and dendrites of cortical neurons transfected with dsRed-Mito and the control, TRAK1-scrRNA, or TRAK1-shRNA at 7 DIV and imaged at 10 DIV. The video is shown at 7 fps. Supplementary Video 3.

Supplementary Video 4 Mitochondrial mobility in axons and dendrites of hippocampal neurons transfected with dsRed-Mito and the control, TRAK1-scrRNA, or TRAK1-shRNA at 11 DIV and imaged at 14 DIV. The video is shown at 7 fps. Supplementary Video 4.

Supplementary Video 5 Mitochondrial mobility in axons and dendrites of cortical neurons transfected with dsRed-Mito and the control, TRAK2-scrRNA, or TRAK2-shRNA at 11 DIV and imaged at 14 DIV. The video is shown at 7 fps. Supplementary Video 5.

Supplementary Video 6 Mitochondrial mobility in axons and dendrites of hippocampal neurons transfected with dsRed-Mito and the control, TRAK2-scrRNA, or TRAK2-shRNA at 11 DIV and imaged at 14 DIV. The video is shown at 7 fps. Supplementary Video 6.

## Conflict of interest

All authors declare they have no conflict of interest.

## Role of authors

All authors had full access to all the data in the study and take responsibility for the integrity of the data and the accuracy of the data analysis. Study concept and design: OL, FAS. Acquisition of data: OL. Analysis and interpretation of data: OL. Drafting of the manuscript: OL. Critical revision of the manuscript for important intellectual content: OL, FAS. Statistical analysis: OL. Obtained funding: FAS. Administrative, technical, and material support: OL, FAS. Study supervision: FAS.

## Funding

This work was supported by the Biotechnology and Biological Sciences Research Council (BBSRC) [grant number BB/K014285/1].

## Figures and Tables

**Table 1:** A summary of mitochondrial mobilities and velocities in axons and dendrites of cortical and hippocampal neurons at different stages of maturation.

Neuronal Type	Compartment	Mobile mitochondria (% ± SEM)				Mitochondrial mean velocity (μm/s ± SEM)			
		N	6 DIV	10 DIV	14 DIV	N	6 DIV	10 DIV	14 DIV
Cortical	Axon	1A	29.6 ± 1.9	28.5 ± 1.5^ns^	25.1 ± 1.8^⁎^	1B	0.39 ± 0.03	0.46 ± 0.04^ns^	0.53 ± 0.04^⁎⁎^
Cortical	Dendrite	2A	20.5 ± 0.9	16.5 ± 1.5^⁎^	16.5 ± 1.6^⁎^	2B	0.39 ± 0.06	0.49 ± 0.03^⁎⁎^	0.53 ± 0.07^⁎⁎^
Hippocampal	Axon	3A	28.4 ± 2.0	27.2 ± 1.1^ns^	24.9 ± 1.4^⁎^	3B	0.50 ± 0.03	0.49 ± 0.04^ns^	0.61 ± 0.01^⁎^
Hippocampal	Dendrite	4A	27.0 ± 1.1	26.9 ± 1.2^ns^	26.9 ± 1.9^ns^	4B	0.43 ± 0.03	0.53 ± 0.05^⁎^	0.61 ± 0.03^⁎^

Cortical and hippocampal neurons were transfected with DsRed-Mito. Transfected neurons were imaged by confocal microscopy at the indicated DIV and the results analyzed using ImageJ as described in Material and methods. Values are means ± SEM from ~ 30 axons or 30 dendrites (i.e. 1000 mitochondria) from 5 independent transfection experiments. Statistical significances were obtained using the Kruskal-Wallis test for each N group followed by the Tukey's range test for samples where significances were found. The results were:- Kruskal-Wallis test ρ: 1A = 0.0484; 2A = 0.03; 3A = 0.045; 4A = ns; 1B = 0.04; 2B = 0.019; 3B = 0.031; 4B = 0.0251. The multiple comparison Tukey's range test between 6 DIV and 10 DIV or 6 DIV and 14 DIV gave values of ^⁎^ρ < 0.05, ^⁎⁎^ρ < 0.01, ^⁎⁎⁎^ρ < 0.001; ns = not significant.

**Table 2:** A summary of mitochondrial anterograde, retrograde and bidirectional movements in axons and dendrites of cortical and hippocampal neurons at different stages of maturation.

Neuronal type	Compartment	Anterograde mitochondria (% ± SEM)			Retrograde mitochondria (% ± SEM)			Bidirectional mitochondria (% ± SEM)		
		6 DIV	10 DIV	14 DIV	6 DIV	10 DIV	14 DIV	6 DIV	10 DIV	14 DIV
Cortical	Axon	43.5 ± 2.4	44.2 ± 2.5^ns^	43.9 ± 3.0^ns^	47.1 ± 2.4	46.2 ± 2.6^ns^	46.0 ± 1.7^ns^	9.4 ± 0.4	9.6 ± 0.3^ns^	10.1 ± 0.5^ns^
Cortical	Dendrite	45.6 ± 1.9	45.6 ± 2.0^ns^	45.5 ± 2.1^ns^	43.4 ± 2.0	44.5 ± 1.8^ns^	43.5 ± 0.9^ns^	11.0 ± 1.0	9.9 ± 0.4^ns^	11.0 ± 0.1^ns^
Hippocampal	Axon	49.9 ± 2.8	44.2 ± 2.0^⁎^	46.8 ± 2.1^ns^	38.0 ± 2.1	42.7 ± 1.7^ns^	41.7 ± 1.2^ns^	12.1 ± 0.1	13.1 ± 0.5^ns^	11.5 ± 1.0^ns^
Hippocampal	Dendrite	41.6 ± 1.0	42.1 ± 4.0^ns^	44.1 ± 1.9^ns^	47.9 ± 2.1	47.8 ± 2.1^ns^	44.4 ± 2.1^ns^	10.5 ± 0.5	10.1 ± 1.2^ns^	11.5 ± 0.8^ns^

Cortical and hippocampal neurons were transfected with DsRed-Mito. Transfected neurons were imaged by confocal microscopy at the indicated DIV and the results analyzed using ImageJ as described in Material and methods. Values are means ± SEM from ~ 30 axons or 30 dendrites (i.e. 1000 mitochondria) from 5 independent transfection experiments. Statistical significances were obtained using the Kruskal-Wallis test followed by the Tukey's range test where appropriate. The results were:- Kruskal-Wallis test ρ: All were not significant except anterograde mitochondria, in hippocampal axons, 10 DIV = 0.041. The multiple comparison Tukey's range test between 6 DIV and 10 DIV or 6 DIV and 14 DIV gave a value of ^⁎^ρ < 0.05; ns = not significant.

**Table 3:** A summary of mitochondrial mobilities and velocities in axons and dendrites of cortical and hippocampal neurons at different stages of maturation: the effect of TRAK1 gene knockdown using TRAK1-shRNA.

Neuronal type	Clones transfected^#^	Compartment	Mobile mitochondria (% ± SEM)			Mitochondrial mean velocity (μm/s ± SEM)		
			6 DIV	10 DIV	14 DIV	6 DIV	10 DIV	14 DIV
			1A	2A	3A	1B	2B	3B
Cortical	–	Axon	29.6 ± 1.9	28.5 ± 1.5	25.1 ± 1.8	0.39 ± 0.03	0.46 ± 0.04	0.53 ± 0.04
Cortical	TRAK1-scrRNA	Axon	29.5 ± 1.8	29.6 ± 1.7	25.5 ± 2.3	0.46 ± 0.03	0.40 ± 0.03	0.46 ± 0.03
Cortical	TRAK1-shRNA	Axon	9.4 ± 1.0^⁎⁎⁎^	9.9 ± 1.4^⁎⁎⁎^	10.3 ± 2.2^⁎⁎⁎^	0.47 ± 0.08^ns^	0.39 ± 0.02^ns^	0.51 ± 0.04^ns^

			4A	5A	6A	4B	5B	6B
Cortical	–	Dendrite	20.5 ± 0.9	16.5 ± 1.5	16.5 ± 1.6	0.39 ± 0.06	0.49 ± 0.03	0.53 ± 0.07
Cortical	TRAK1-scrRNA	Dendrite	20.7 ± 1.2	16.2 ± 1.6	18.4 ± 1.6	0.40 ± 0.05	0.52 ± 0.06	0.52 ± 0.03
Cortical	TRAK1-shRNA	Dendrite	13.9 ± 1.4^⁎⁎^	14.0 ± 1.3^ns^	15.0 ± 2.6^ns^	0.42 ± 0.03^ns^	0.55 ± 0.05^ns^	0.54 ± 0.06^ns^

			7A	8A	9A	7B	8B	9B
Hippocampal	–	Axon	28.4 ± 2.0	27.2 ± 1.1	24.9 ± 1.4	0.50 ± 0.03	0.49 ± 0.04	0.61 ± 0.01
Hippocampal	TRAK1-scrRNA	Axon	28.0 ± 2.3	28.2 ± 2.6	25.9 ± 2.3	0.51 ± 0.03	0.47 ± 0.04	0.59 ± 0.05
Hippocampal	TRAK1-shRNA	Axon	13.9 ± 1.7^⁎⁎⁎^	13.9 ± 1.4^⁎⁎⁎^	12.0 ± 1.3^⁎⁎^	0.53 ± 0.03^ns^	0.55 ± 0.05^ns^	0.55 ± 0.06^ns^

			10A	11A	12A	10B	11B	12B
Hippocampal	–	Dendrite	27.0 ± 1.1	26.9 ± 1.2	26.9 ± 1.9	0.43 ± 0.03	0.53 ± 0.05	0.61 ± 0.03
Hippocampal	TRAK1-scrRNA	Dendrite	25.5 ± 2.3	27.0 ± 2.7	27.1 ± 2.2	0.42 ± 0.03	0.53 ± 0.04	0.60 ± 0.05
Hippocampal	TRAK1-shRNA	Dendrite	9.9 ± 2.1^⁎⁎^	12.5 ± 2.1^⁎⁎^	19.5 ± 3.0^ns^	0.53 ± 0.05^ns^	0.43 ± 0.04^ns^	0.58 ± 0.11^ns^

Cortical and hippocampal neurons were transfected with either DsRed-Mito (−), DsRed-Mito + TRAK1-scrRNA or DsRed-Mito + TRAK1-shRNA. Transfected neurons were imaged by confocal microscopy at the indicated DIV and the results analyzed using ImageJ as described in Material and methods. Values are means ± SEM from ~ 30 axons or 30 dendrites (i.e. 1000 mitochondria) from 5 independent transfection experiments. Statistical significances were obtained using the Kruskal-Wallis test followed by the Tukey's range test for each group followed by the Tukey's range test for samples where significances were found. The results were:- Kruskal-Wallis test ρ: 1A–2A–3A–7A–8A–9A ≤ 0.001; 4A = 0.0013; 10A = 0.005; 11A = 0.0023; 5A–6A–12A = ns; All B samples were ns. The multiple comparison Tukey's range test between TRAK1-scrRNA and TRAK1-shRNA gave values ^⁎⁎^ρ < 0.01, ^⁎⁎⁎^ρ < 0.001; ns = not significant.

**Table 4:** A summary of mitochondrial anterograde, retrograde and bidirectional movements in axons and dendrites of cortical and hippocampal neurons at different stages of maturation: the effect of TRAK1 gene knockdown using TRAK1-shRNA.

Neuronal type	Clones transfected^#^	Compartment	Anterograde mitochondria (% ± SEM)			Retrograde mitochondria (% ± SEM)			Bidirectional mitochondria (% ± SEM)		
			6 DIV	10 DIV	14 DIV	6 DIV	10 DIV	14 DIV	6 DIV	10 DIV	14 DIV
Cortical	–	Axon	43.5 ± 2.4	44.2 ± 2.5	43.9 ± 3.0	47.1 ± 2.4	46.2 ± 2.6	46.0 ± 1.7	9.4 ± 0.4	9.6 ± 0.3	10.1 ± 0.5
Cortical	TRAK1-scrRNA	Axon	44.1 ± 1.0	44.1 ± 3.8	45.5 ± 2.1	46.4 ± 0.8	44.9 ± 1.7	43.4 ± 2.2	9.5 ± 1.2	11.0 ± 1.8	11.1 ± 1.0
Cortical	TRAK1-shRNA	Axon	42.1 ± 1.4^ns^	43.0 ± 1.2^ns^	45.0 ± 1.6^ns^	49.0 ± 2.1^ns^	48.0 ± 1.3^ns^	44.7 ± 1.8^ns^	8.9 ± 1.5^ns^	9.0 ± 1.3^ns^	10.3 ± 0.7^ns^
Cortical	–	Dendrite	45.6 ± 1.9	45.6 ± 2.0	45.5 ± 2.1	43.4 ± 2.0	44.5 ± 1.8	43.5 ± 0.9	11.0 ± 1.0	9.9 ± 0.4	11.0 ± 0.1
Cortical	TRAK1-scrRNA	Dendrite	42.6 ± 0.9	42.6 ± 1.6	44.1 ± 2.9	47.3 ± 2.1	47.2 ± 0.8	43.7 ± 0.7	10.1 ± 0.5	10.2 ± 1.3	12.2 ± 1.5
Cortical	TRAK1-shRNA	Dendrite	46.5 ± 2.5^ns^	45.6 ± 0.3^ns^	44.9 ± 2.0^ns^	44.6 ± 2.4^ns^	44.2 ± 0.7^ns^	45.0 ± 2.8^ns^	8.9 ± 1.3^ns^	10.2 ± 1.1^ns^	10.1 ± 0.9^ns^
Hippocampal	–	Axon	49.9 ± 2.8	44.2 ± 2.0	46.8 ± 2.1	38.0 ± 2.1	42.7 ± 1.7	41.7 ± 1.2	12.1 ± 0.1	13.1 ± 0.5	11.5 ± 1.0
Hippocampal	TRAK1-scrRNA	Axon	39.8 ± 3.4	41.9 ± 2.8	42.5 ± 1.9	48.7 ± 0.7	48.2 ± 1.8	47.5 ± 2.0	11.5 ± 2.2	9.9 ± 2.0	10.0 ± 1.3
Hippocampal	TRAK1-shRNA	Axon	44.5 ± 1.9^⁎^	43.5 ± 2.0^ns^	42.5 ± 2.5^ns^	44.5 ± 3.0^ns^	46.1 ± 3.0^ns^	46.5 ± 2.5^ns^	11.0 ± 1.3^ns^	10.4 ± 1.4^ns^	11.0 ± 0.3^ns^
Hippocampal	–	Dendrite	41.6 ± 1.0	42.1 ± 4.0	44.1 ± 1.9	47.9 ± 2.1	47.8 ± 2.1	44.4 ± 2.1	10.5 ± 0.5	10.1 ± 1.2	11.5 ± 0.8
Hippocampal	TRAK1-scrRNA	Dendrite	41.6 ± 2.4	42.0 ± 1.8	42.1 ± 2.9	48.3 ± 1.9	48.2 ± 2.6	46.9 ± 1.1	10.1 ± 1.2	9.8 ± 1.1	9.9 ± 0.2
Hippocampal	TRAK1-shRNA	Dendrite	41.6 ± 1.7^ns^	44.5 ± 1.6^ns^	45.6 ± 0.2^ns^	48.2 ± 2.7^ns^	44.8 ± 0.8^ns^	44.3 ± 4.9^ns^	10.2 ± 1.8^ns^	10.7 ± 1.4^ns^	10.1 ± 1.0^ns^

– # All samples transfected also with DsRed-Mito.

Cortical and hippocampal neurons were transfected with either DsRed-Mito (−), DsRed-Mito + TRAK1-scrRNA or DsRed-Mito + TRAK1-shRNA. Transfected neurons were imaged by confocal microscopy at the indicated DIV and the results analyzed using ImageJ as described in Material and methods. Values are means ± SEM from ~ 30 axons or 30 dendrites (i.e. 1000 mitochondria) from 5 independent transfection experiments. Statistical significances were obtained using the Kruskal-Wallis test followed by the Tukey's range test for each group for samples where significances were found. The results were:- Kruskal-Wallis test, all were not significant except for anterograde mitochondria in hippocampal axons at 6 DIV ρ = 0.025. The multiple comparison Tukey's range test between TRAK1-scrRNA and TRAK1-shRNA for 6 DIV anterograde axonal mitochondrial transport gave a ^⁎^ρ < 0.05; ns = not significant.

**Table 5:** A summary of mitochondrial mobilities and velocities in axons and dendrites of cortical and hippocampal neurons at different stages of maturation: the effect of TRAK2 gene knockdown using TRAK2-shRNA.

Neuronal type	Clones transfected^#^	Compartment	Mobile mitochondria (% ± SEM)			Mitochondrial mean velocity (μm/s ± SEM)		
			6 DIV	10 DIV	14 DIV	6 DIV	10 DIV	14 DIV
			1A	2A	3A	1B	2B	3B
Cortical	–	Axon	29.6 ± 1.9	28.5 ± 1.5	25.1 ± 1.8	0.39 ± 0.03	0.46 ± 0.04	0.53 ± 0.04
Cortical	TRAK2-scrRNA	Axon	33.3 ± 2.6	25.6 ± 1.6	25.0 ± 3.9	0.46 ± 0.03	0.40 ± 0.05	0.46 ± 0.05
Cortical	TRAK2-shRNA	Axon	21.7 ± 2.0^⁎^	18.6 ± 1.3^⁎^	27.1 ± 1.8^ns^	0.47 ± 0.08^ns^	0.39 ± 0.01^ns^	0.51 ± 0.05^ns^

			4A	5A	6A	4B	5B	6B
Cortical	–	Dendrite	20.5 ± 0.9	16.5 ± 1.5	16.5 ± 1.6	0.39 ± 0.06	0.49 ± 0.03	0.53 ± 0.07
Cortical	TRAK2-scrRNA	Dendrite	18.8 ± 2.0	15.8 ± 1.7	14.9 ± 0.6	0.40 ± 0.06	0.52 ± 0.05	0.52 ± 0.05
Cortical	TRAK2-shRNA	Dendrite	8.8 ± 1.4^⁎⁎^	10.2 ± 1.0^⁎⁎^	11.1 ± 1.6^⁎^	0.42 ± 0.04^ns^	0.55 ± 0.08^ns^	0.54 ± 0.06^ns^

			7A	8A	9A	7B	8B	9B
Hippocampal	–	Axon	28.4 ± 2.0	27.2 ± 1.1	24.9 ± 1.4	0.50 ± 0.03	0.49 ± 0.04	0.61 ± 0.01
Hippocampal	TRAK2-scrRNA	Axon	24.5 ± 2.7	24.5 ± 3.0	27.6 ± 4.1	0.51 ± 0.06	0.47 ± 0.06	0.59 ± 0.06
Hippocampal	TRAK2-shRNA	Axon	17.9 ± 2.4^ns^	15.9 ± 2.5^⁎^	22.9 ± 1.6^ns^	0.53 ± 0.04^ns^	0.55 ± 0.07^ns^	0.55 ± 0.07^ns^

			10A	11A	12A	10B	11B	12B
Hippocampal	–	Dendrite	27.0 ± 1.1	26.9 ± 1.2	26.9 ± 1.9	0.43 ± 0.03	0.53 ± 0.05	0.61 ± 0.03
Hippocampal	TRAK2-scrRNA	Dendrite	22.3 ± 1.6	24.7 ± 2.7	25.0 ± 2.4	0.42 ± 0.04	0.53 ± 0.05	0.60 ± 0.05
Hippocampal	TRAK2-shRNA	Dendrite	12.7 ± 2.3^⁎^	10.8 ± 1.6^⁎^	17.6 ± 1.4^⁎^	0.53 ± 0.04^ns^	0.43 ± 0.05^ns^	0.58 ± 0.10^ns^

– # All samples transfected also with DsRed-Mito.

Cortical and hippocampal neurons were transfected with either DsRed-Mito (−), DsRed-Mito + TRAK2-scrRNA or DsRed-Mito + TRAK2-shRNA. Transfected neurons were imaged by confocal microscopy at the indicated DIV and the results analyzed using ImageJ as described in Material and methods. Values are means ± SEM from ~ 30 axons or 30 dendrites (i.e. 1000 mitochondria) from 5 independent transfection experiments. Statistical significances were obtained using the Kruskal-Wallis test followed by the Tukey's range test for each group for samples where significances were found. The results were:- Kruskal-Wallis test ρ: 1A = 0.0047; 2A = 0.0015; 4A = 0.001; 5A = 0.0049; 8A = 0.0062; 10A = 0.0132; 11A = 0.005; 3A-6A-7A-9A-12A = ns; All B velocity values were not significant. The multiple comparison Tukey's range test between TRAK2-scrRNA and TRAK2-shRNA gave *p* values as shown with ^⁎^ρ < 0.05, ^⁎⁎^ρ < 0.01; ns = not significant.

**Table 6:** A summary of mitochondrial anterograde, retrograde and bidirectional movements in axons and dendrites of cortical and hippocampal neurons at different stages of maturation: the effect of TRAK2 gene knockdown using TRAK2-shRNA.

Neuronal type	Clones transfected^#^	Compartment	Anterograde mitochondria (% ± SEM)			Retrograde mitochondria (% ± SEM)			Bidirectional mitochondria (% ± SEM)		
			6 DIV	10 DIV	14 DIV	6 DIV	10 DIV	14 DIV	6 DIV	10 DIV	14 DIV
Cortical	–	Axon	43.5 ± 2.4	44.2 ± 2.5	43.9 ± 3.0	47.1 ± 2.4	46.2 ± 2.6	46.0 ± 1.7	9.4 ± 0.4	9.6 ± 0.3	10.1 ± 0.5
Cortical	TRAK2-scrRNA	Axon	46.5 ± 2.5	45.6 ± 2.5	45.1 ± 1.8	44.1 ± 1.6	45.0 ± 2.0	44.5 ± 1.8	9.4 ± 0.2	9.4 ± 1.9	10.4 ± 0.9
Cortical	TRAK2-shRNA	Axon	46.1 ± 2.0^ns^	46.0 ± 1.6^ns^	44.9 ± 1.9^ns^	44.5 ± 1.9^ns^	44.6 ± 1.0^ns^	44.7 ± 1.5^ns^	7.5 ± 1.2^⁎^	7.9 ± 1.6^ns^	8.9 ± 1.4^ns^
Cortical	–	Dendrite	45.6 ± 1.9	45.6 ± 2.0	45.5 ± 2.1	43.4 ± 2.0	44.5 ± 1.8	43.5 ± 0.9	11.0 ± 1.0	9.9 ± 0.4	11.0 ± 0.1
Cortical	TRAK2-scrRNA	Dendrite	42.0 ± 2.2	44.1 ± 1.6	50.0 ± 2.6	48.1 ± 2.1	46.0 ± 2.1	39.7 ± 1.8	9.9 ± 1.0	9.9 ± 1.0	10.3 ± 1.4
Cortical	TRAK2-shRNA	Dendrite	42.0 ± 1.8^ns^	42.6 ± 2.0^ns^	47.8 ± 3.0^ns^	47.8 ± 1.9^ns^	47.1 ± 0.9^ns^	42.4 ± 2.7^ns^	10.2 ± 1.8^ns^	10.3 ± 1.3^ns^	9.8 ± 1.6^ns^
Hippocampal	–	Axon	49.9 ± 2.8	44.2 ± 2.0	46.8 ± 2.1	38.0 ± 2.1	42.7 ± 1.7	41.7 ± 1.2	12.1 ± 0.1	13.1 ± 0.5	11.5 ± 1.0
Hippocampal	TRAK2-scrRNA	Axon	44.6 ± 2.8	44.6 ± 2.7	45.6 ± 1.9	48.6 ± 3.1	43.0 ± 1.9	44.3 ± 1.8	6.8 ± 2.0	12.4 ± 2.3	10.1 ± 0.9
Hippocampal	TRAK2-shRNA	Axon	43.9 ± 3.7^ns^	39.9 ± 8.1^⁎^	43.8 ± 4.8^ns^	45.4 ± 8.8^ns^	49.4 ± 7.8^⁎⁎^	45.9 ± 2.6^ns^	10.7 ± 5.1^⁎^	10.7 ± 1.9^ns^	10.3 ± 3.0^ns^
Hippocampal	–	Dendrite	41.6 ± 1.0	42.1 ± 4.0	44.1 ± 1.9	47.9 ± 2.1	47.8 ± 2.1	44.4 ± 2.1	10.5 ± 0.5	10.1 ± 1.2	11.5 ± 0.8
Hippocampal	TRAK2-scrRNA	Dendrite	42.8 ± 2.4	45.0 ± 2.7	43.9 ± 2.0	45.1 ± 2.9	42.8 ± 1.7	44.6 ± 2.5	12.1 ± 1.9	12.2 ± 0.8	11.5 ± 1.7
Hippocampal	TRAK2-shRNA	Dendrite	43.8 ± 1.9^ns^	45.0 ± 1.7^ns^	44.1 ± 2.1^ns^	44.2 ± 1.1^ns^	43.0 ± 2.1^ns^	46.1 ± 0.4^ns^	12.0 ± 1.8^ns^	12.0 ± 1.7^ns^	9.8 ± 1.1^ns^

– # All samples transfected also with DsRed-Mito.

Cortical and hippocampal neurons were transfected with either DsRed-Mito (−), DsRed-Mito + TRAK2-scrRNA or DsRed-Mito + TRAK2-shRNA. Transfected neurons were imaged by confocal microscopy at the indicated DIV and the results analyzed using ImageJ as described in Material and methods. Values are means ± SEM from ~ 30 axons or 30 dendrites (i.e. 1000 mitochondria) from 5 independent transfection experiments. Statistical significances were obtained using the Kruskal-Wallis test followed by the Tukey's range test for each group for samples where significances were found. The results were:- Kruskal-Wallis test, anterograde mitochondria, hippocampal axon, 10 DIV = 0.031; retrograde mitochondria, hippocampal axon, 10 DIV = 0.0123; bidirectional mitochondria, cortical axon, 6 DIV = 0.028; bidirectional mitochondria, hippocampal axon, 6 DIV = 0.0358; all other vales were not significant. The multiple comparison Tukey's range test between TRAK2-scrRNA and TRAK2-shRNA gave *p* values as shown with ^⁎^ρ < 0.05; ns = not significant.
