# Supplementary figures and images for: Developmental changes in trak-mediated mitochondrial transport in neurons
Source: Mol Cell Neurosci. 2017 Apr;80:134–47. doi: 10.1016/j.mcn.2017.03.006 (PMC5400476; doi:10.1016/j.mcn.2017.03.006)

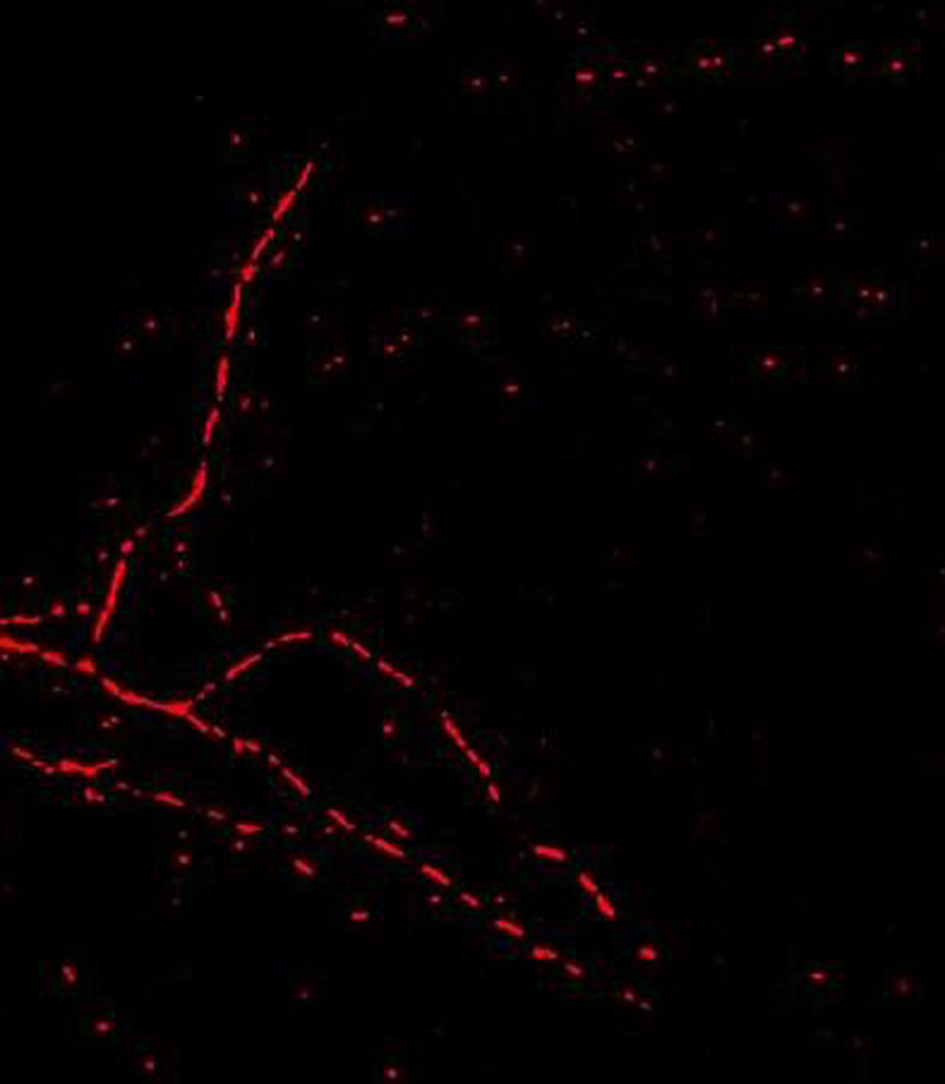

Supplement: Supplementary Video 1 — An example of mitochondrial dynamics in axons of hippocampal neurons. Neurons were transfected with dsRed-Mito at 7 DIV. Time lapsed images were obtained of living neurons at 10 DIV capturing an image every 3 s for a total of 5 min (total images = 100). The video is shown at 7 frames per second (fps). [file mmc1.jpg]

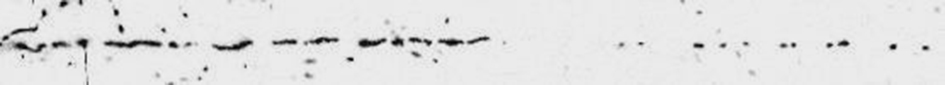

Supplement: Supplementary Video 2 — An example of straightened axons from Supplementary Video 1 for analysis using ImageJ plugin Straighten. The video is shown at 7 fps. [file mmc2.jpg]

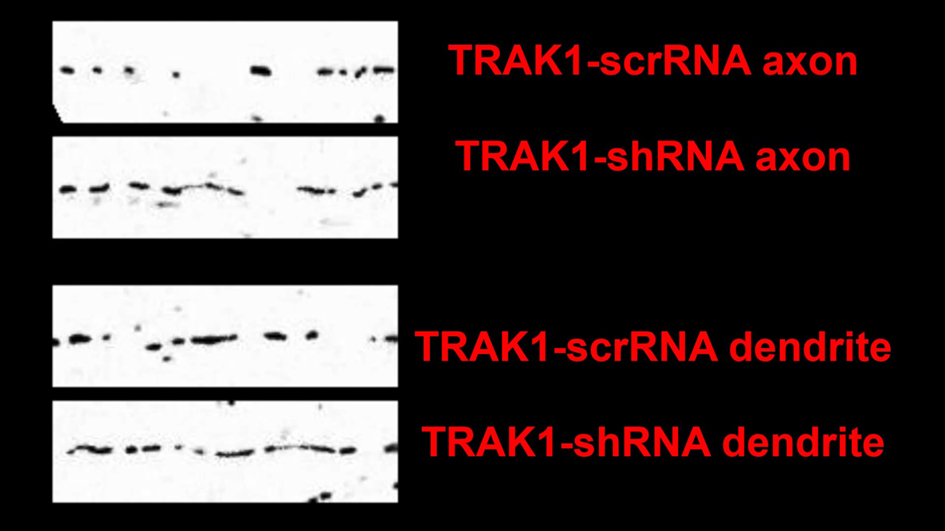

Supplement: Supplementary Video 3 — Mitochondrial mobility in axons and dendrites of cortical neurons transfected with dsRed-Mito and the control, TRAK1-scrRNA, or TRAK1-shRNA at 7 DIV and imaged at 10 DIV. The video is shown at 7 fps. [file mmc3.jpg]

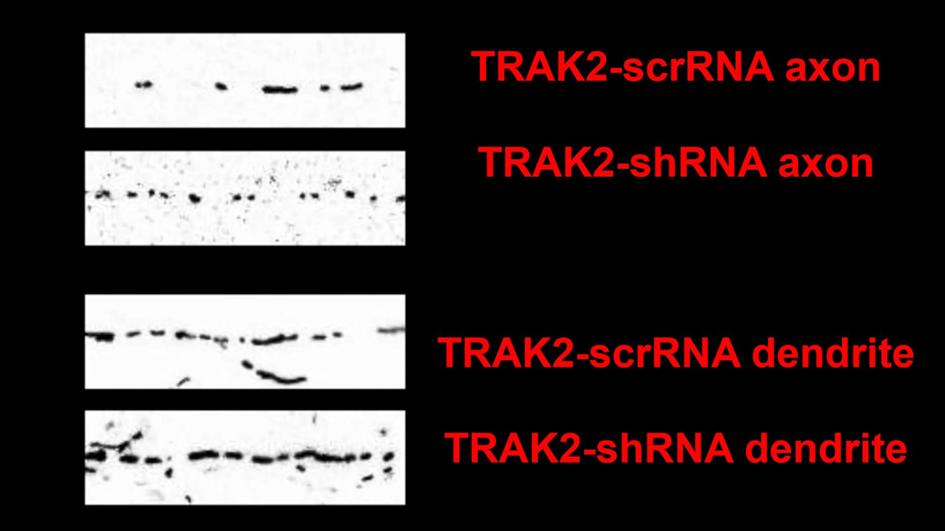

Supplement: Supplementary Video 4 — Mitochondrial mobility in axons and dendrites of hippocampal neurons transfected with dsRed-Mito and the control, TRAK1-scrRNA, or TRAK1-shRNA at 11 DIV and imaged at 14 DIV. The video is shown at 7 fps. [file mmc4.jpg]

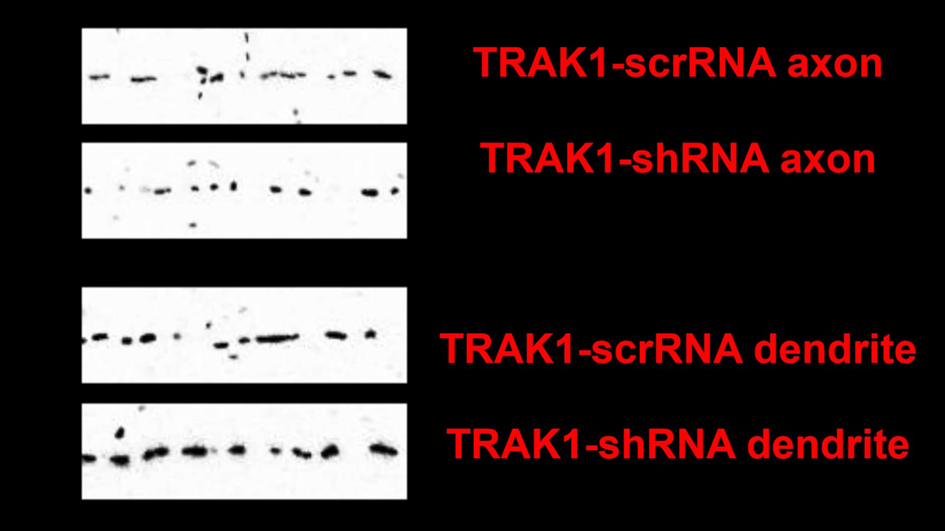

Supplement: Supplementary Video 5 — Mitochondrial mobility in axons and dendrites of cortical neurons transfected with dsRed-Mito and the control, TRAK2-scrRNA, or TRAK2-shRNA at 11 DIV and imaged at 14 DIV. The video is shown at 7 fps. [file mmc5.jpg]

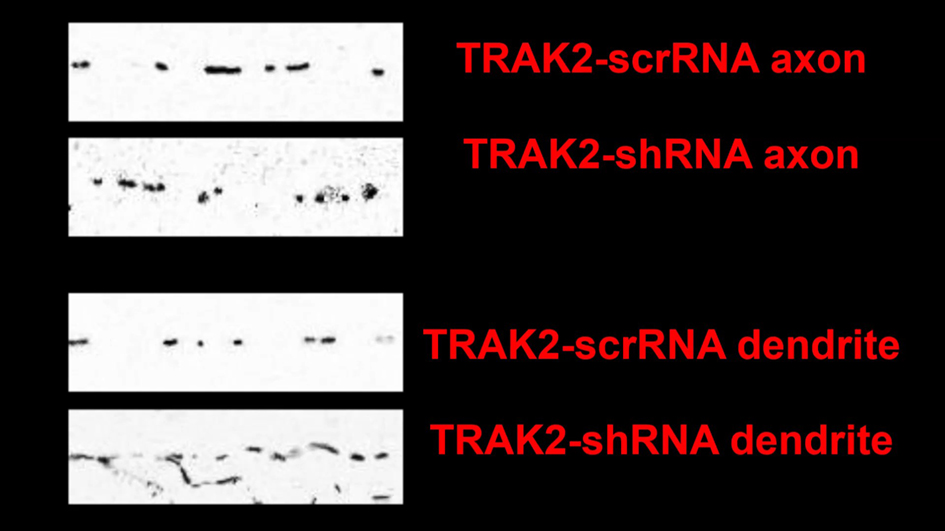

Supplement: Supplementary Video 6 — Mitochondrial mobility in axons and dendrites of hippocampal neurons transfected with dsRed-Mito and the control, TRAK2-scrRNA, or TRAK2-shRNA at 11 DIV and imaged at 14 DIV. The video is shown at 7 fps. [file mmc6.jpg]
